# TIME IS OF THE ESSENCE: RELIABLE MEASUREMENT OF HEART RATE VARIABILITY IN OLDER ADULTS

**DOI:** 10.1093/geroni/igac059.2413

**Published:** 2022-12-20

**Authors:** Cristina Pinheiro, Suzanne Segerstrom, Justin Karr

**Affiliations:** University of Kentucky, Lexington, Kentucky, United States; University of Kentucky, Lexington, Kentucky, United States; University of Kentucky, Lexington, Kentucky, United States

## Abstract

Heart rate variability (HRV) decreases with age and is an important correlate of psychosocial and physical health. Recommendations for the minimum duration of EKG to accurately derive HRV vary from 1 minute to several minutes. However, the definition of “accuracy” or reliability depends on study design, including whether the focus of the study is on stable or momentary between-person differences or within-person changes in HRV. In a sample of 216 older adults (Mage = 72.7, 62.5% women), ECG was measured at 1000 Hz for 10 minutes every 6 months for up to 6.5 years. HRV was high-frequency power (0.15–0.40). A generalizability study determined the variance due to minute, occasion, person, and their interactions. The most variance was due to idiosyncratic occasion differences (46%), followed by stable person variance (18%). A decision study determined how accuracy in HRV measurement could be achieved. Between-person differences in HRV at a specific occasion could be reliably measured with 6 minutes of ECG (.80); within-person changes in HRV between occasions could be reliably measured with 3 minutes of ECG (.83). For stable individual differences, 10 or more occasions of 1-minute duration produced a more reliable estimate (.73) than increasing the length of a single ECG recording to 10 minutes (.25). The necessary duration of ECG for measuring HRV reliably depends on the study design and research question. Ultra-short durations (1-2 minutes) are generally not recommended for older adults.

